# A Case of Severe Dilated Cardiomyopathy and Hyperthyroidism

**DOI:** 10.7759/cureus.22968

**Published:** 2022-03-08

**Authors:** Mohammad Haidous, Abdul Rahman Al Armashi, Patil Balozian, Keyvan Ravakhah

**Affiliations:** 1 Internal Medicine, St. Vincent Charity Medical Center, Cleveland, USA

**Keywords:** hyperthyroid cardiomyopathy, reversible cardiomyopathy, dilated cardiomyopathy, acute heart failure, hyperthyroidism

## Abstract

Hyperthyroidism often leads to heart failure when left untreated, specifically high output heart failure and left ventricular (LV) hypertrophy. A very minimal portion of those develop severe LV dysfunction. We report a case of a 65-year-old male who presented with signs and symptoms of heart failure and was found to have hyperthyroidism, severe systolic dysfunction, and severe dilated cardiomyopathy.

The patient is a 65-year-old African American male with a history of hypertension (HTN) who presented with complaints of dyspnea on exertion and bilateral lower limb edema of one-week duration. A review of systems revealed paroxysmal nocturnal dyspnea, orthopnea, palpitations, fatigue, and weight loss. Physical exam showed tachycardia but otherwise no exophthalmos, no thyromegaly, no thyroid nodules, clear lungs, normal heart sounds, regular heart rhythm, normal reflexes, and 2+ edema of bilateral lower extremities up to the knees. Labs showed elevated B-natriuretic peptide, severely suppressed thyroid-stimulating hormone, elevated free triiodothyronine (FT3), and free thyroxine (FT4). Electrocardiogram (EKG) revealed sinus tachycardia, incomplete left bundle branch block, and non-specific T wave abnormality. Echocardiography revealed abnormal (LV) structure and function, with moderate to severe dilatation without LV hypertrophy, severe LV systolic dysfunction with ejection fraction (EF) 30-35%, and an abnormal LV diastolic function.

The patient was managed with diuresis for acute onset heart failure and with beta-blocker and methimazole for symptomatic hyperthyroidism.

Thyroid assessment is an important step in evaluating any patient with suspected heart failure. This case highlights the balance that should exist between treating hyperthyroidism symptoms and managing disease states such as acute heart failure.

## Introduction

Thyrotoxicosis is defined as a clinical state that ensues due to high thyroid hormone levels exerting their effect on tissues. Hyperthyroidism is a form of thyrotoxicosis that is due to inappropriately elevated synthesis and secretion of thyroid hormones by the thyroid gland [1,2]. In the US, the prevalence of hyperthyroidism is 1.2% of which 0.7% are subclinical and 0.5% are overt. The causes are variable and include Grave’s disease, toxic multinodular goiter, painless transient thyroiditis, and toxic adenoma [2,3]. Hyperthyroidism manifests itself clinically in a range of symptoms owing to the activation of multiple systems through increased cell surface beta-adrenergic receptors. Symptoms include but are not limited to palpitations, heat intolerance, tremors, proximal muscle weakness, weight loss, and anxiety. In some cases, atrial fibrillation develops (10-15% of patients) or heart failure (5.8% of patients) [2,3]. In this article, we present the case of a 65-year-old male who presented with signs and symptoms of heart failure and was found to have hyperthyroidism.

## Case presentation

**Figure 1:**
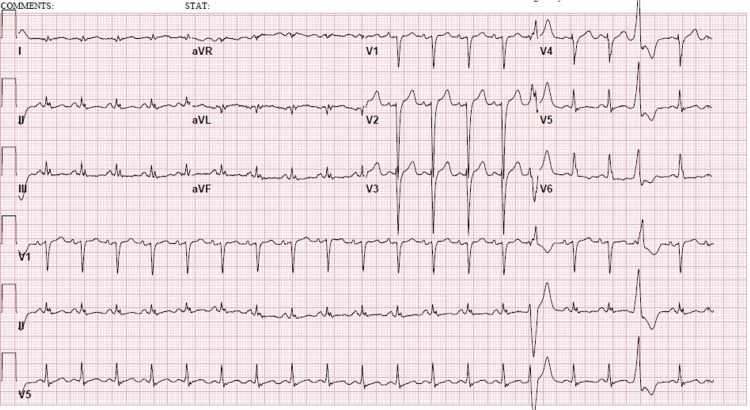
Electrocardiogram showing sinus tachycardia, incomplete left bundle branch block, and non-specific T wave abnormality.

**Figure 2:**
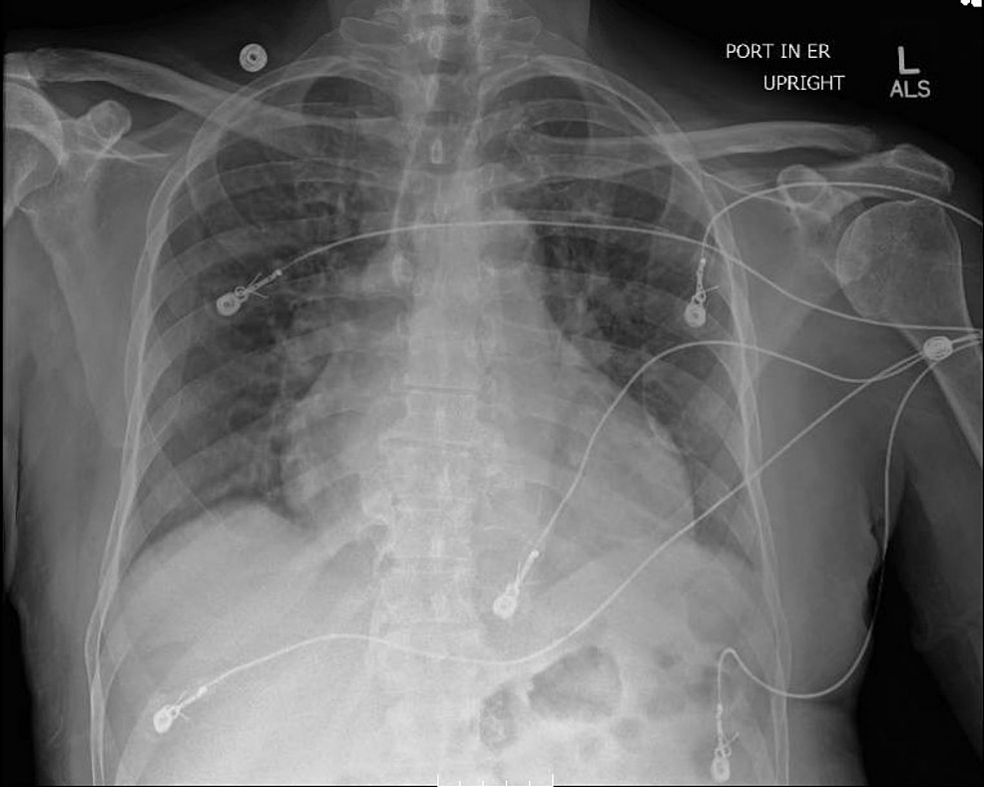
Chest X-ray (Portable)

**Figure 3:**
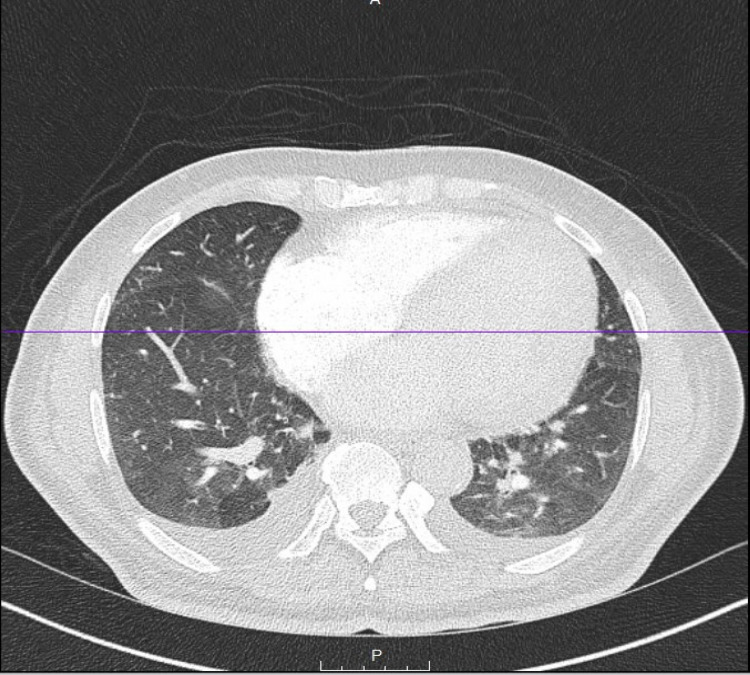
Chest CT angiogram (CTA)

The patient is a 65-year-old African American male with a history of hypertension (HTN) and normal thyroid studies (thyroid-stimulating hormone (TSH), free triiodothyronine (FT3), and free thyroxine (FT4)) two years prior to this presentation who presented with complaints of exertional dyspnea and bilateral lower limb edema of one-week duration. Review of systems revealed 1-2 weeks duration of paroxysmal nocturnal dyspnea, orthopnea, palpitations, and fatigue. He also reported watery, non-bloody diarrhea, and weight loss of around 20 pounds (~10% of body weight) over the last two months. The patient had been on lisinopril, aspirin, atorvastatin, and metoprolol. Vitals showed elevated BP 143/81 mm of Hg, tachycardia with a pulse of 125 beats per minute, otherwise unremarkable with a body mass index of 23.3 kg/m². Physical exam revealed no exophthalmos, a supple neck, no thyromegaly, no thyroid nodules, no distal tremor, clear lungs, normal heart sounds, regular heart rhythm, tachycardia, normal reflexes, and 2+ edema of bilateral lower extremities up to the knees with no calf tenderness. Labs showed normocytic anemia, hemoglobin 12.4 g/dL with a mean corpuscular volume (MCV) of 92 fL, and a normal complete metabolic panel. Iron studies showed normal serum iron, total iron-binding capacity (TIBC), and ferritin. Troponin was negative x3 times, 0.03ng/mL, 0.033 ng/mL, and 0.017 ng/mL. The B-natriuretic peptide was elevated to 1238.34 pg/mL, the TSH was <0.005 uIU/mL, FT3 was 5.38 pg/mL, and FT4 was 2.25 ng/dL (Table 1). Electrocardiogram (EKG) revealed sinus tachycardia, incomplete left bundle branch block, and non-specific T wave abnormality (Figure 1). Chest X-ray revealed an enlarged cardiac silhouette (Figure 2). The patient had a chest CT angiogram (CTA) in the emergency department that had revealed small bilateral pleural effusions and left ventriculomegaly (Figure 3, Video 1). Echocardiography revealed abnormal (LV) structure and function, with moderate to severe dilatation without LV hypertrophy, severe LV systolic dysfunction with ejection fraction (EF) 30-35%, and an abnormal LV diastolic function (Videos 2, 3).

**Table 1:** Laboratory Work BUN: Blood urea nitrogen; TIBC: Total iron-binding capacity.

Laboratory work	Value	Reference range
Hemoglobin (g/dL)	12.4	14.0-16.5
Mean Corpuscular Volume (MCV) (fL)	92	80-100
Creatinine (g/dL)	0.934	0.70-1.30
BUN (mg/dL)	12	7-18
Albumin (g/dL)	3.4	2.9-4.4
Troponin (ng/mL)	0.03, 0.033, 0.017	<0.045
B-natriuretic peptide (ng/mL)	1238.34	<100
Thyroid Stimulating Hormone (TSH) (mIU/mL)	<0.005	0.358-3.74
Free triiodothyronine (FT3) (pg/mL)	5.38	2.18-3.98
Free thyroxine (FT4) (ng/dL)	2.25	0.76-1.46
Iron (ug/dL)	69	65-175
TIBC (ug/dL)	250	250-450
Ferritin (ng/mL)	382	26-388
Iron saturation (%)	27	25-35

**Video 1:** Chest CT angiogram (CTA)

**Video 2:** Echocardiogram View 1

**Video 3:** Echocardiogram View 2

The patient was started on intravenous furosemide, and methimazole 10 mg daily. He remained tachycardic despite the metoprolol with a heart rate in the low 100s (110-120). He was closely monitored and on day 3 of admission, the patient had resolution of his bilateral lower limb edema with significant improvement in his dyspnea on exertion, paroxysmal nocturnal dyspnea, and orthopnea. Furosemide was switched to oral form and metoprolol was subsequently increased once the heart failure was deemed to be improving. Following that, his heart rate improved and remained below 100 beats per minute. Endocrinology and cardiology were both consulted for close outpatient follow-up. Unfortunately, the patient was lost to follow-up.

## Discussion

Triiodothyronine (T3) acts on cardiac tissue through genomic and nongenomic pathways. Genomic pathways include binding of T3 with nuclear receptors that have a direct effect on activation or repression of gene expression, thereby regulating the expression of certain proteins. Nongenomic pathways include changes to the myocyte plasma membrane by alterations in sodium, potassium, and calcium ion channels, and changes in cytoskeleton polymerization [1,4]. There also is a role for catecholamines and the Renin-Angiotensin-Aldosterone System in contributing to cardiac effects in abnormal thyroid states [4,5].

Heart failure is a known complication of hyperthyroidism and is usually the presenting sign of hyperthyroidism in around 6% of patients [6-8]. Hyperthyroidism typically causes high output heart failure and LV hypertrophy; less commonly, it causes dilated cardiomyopathy and reduced LV systolic function, as noted in this case [1,7,9]. The number of patients who develop severe LV dysfunction is low at <1% [6]. Several cases have been reported in the literature that demonstrate the reversibility of dilated cardiomyopathy associated with hyperthyroidism, even in severe cases [7,10,11]. In one study, dilated cardiomyopathy associated with hyperthyroidism was completely reversible in 71% of patients included, with significant improvement observed in the remaining 29% [11].

Another study recruited 2,225 patients across the United States, Canada, and New Zealand who suffered from systolic heart failure and were followed for five years. Of those, 13% had abnormal TSH levels at baseline. The study found that abnormal thyroid function in the setting of heart failure increases mortality by about 60% when compared to heart failure in euthyroid patients [12].

Another complication of hyperthyroidism that was manifested in the patient discussed in this case is normochromic, normocytic anemia. Red blood cell mass and plasma volume are both increased in hyperthyroidism, but the latter is increased more, hence leading to normocytic, normochromic anemia. In anemic patients and upon receiving treatment, hemoglobin rises by an average of 0.5 g/dL [13].

## Conclusions

This case highlights the importance of thyroid assessment in patients presenting with heart failure, and the possibility of reversibility of the severe cardiac dysfunction. Hyperthyroidism left undiagnosed and untreated may lead to heart failure, and in a smaller number of patients to severely reduced ejection fraction. It also allows us to appreciate the balance between administering medications, such as beta-blockers, aimed at reducing hyperthyroid symptoms, with the management of heart failure, where beta-blockers might need to be held or administered at regular doses without escalation.
